# Supplementary material for: Development of a Competitive Enzyme-Linked Immunosorbent Assay Targeting the-p30 Protein for Detection of Antibodies against African Swine Fever Virus
Source: Viruses. 2023 Jan 4;15(1):154. doi: 10.3390/v15010154 (PMC9861063; doi:10.3390/v15010154)
Supplement: Supplementary file 1 [file viruses-15-00154-s001.zip › viruses-2132418-supplementary.pdf]

viruses

Supplementary Materials

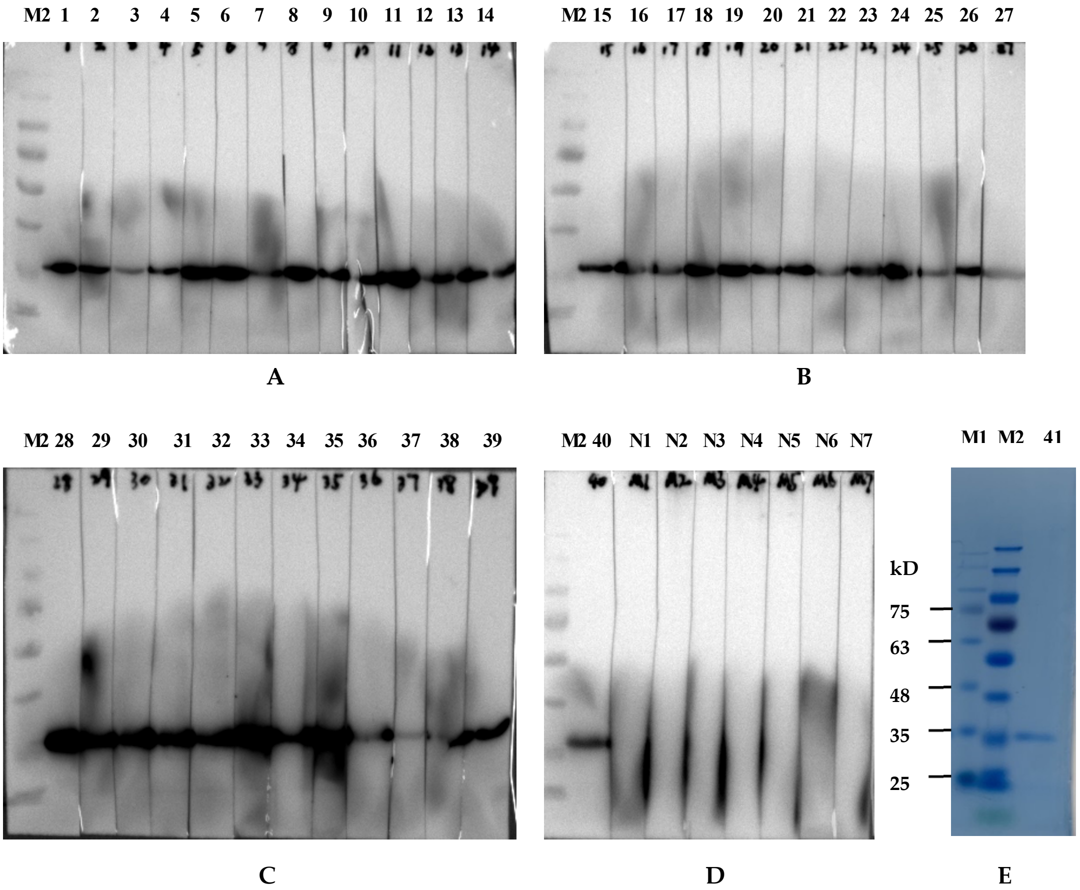

Figure S1. Specificity test of ASFV-positive and -negative sera with purified recombinant p30 (0.4 µg /lane). Western blot of the field samples used as serum panel: **(A)**, **(B)**, **(C)**, **(D)** Purified p30 were diluted to o.4 µg per lane (lane 1–40 and lane N1–N7) in gel electrophoresis, and tested separately with 1000-fold diluted 40 ASFV-positive sera against ASFV. Then, 98 ASFV-negative sera were divided into 7 fractions (N1, N2, N3, N4, N5, N6, N7), and 14 sera in each fraction were tested together with the p30 protein. There were 1 to 40-40 ASFV-positive serum samples, and N1 to N7-7 fractions with 14 ASFV-negative serum samples in each fraction. **(E)** SDS-PAGE analysis of p30 protein. M1—protein ladder purchased from Genedirex, M2—protein ladder purchased from Thermo Fisher, 41—purified p30 (0.4 µg). Ten sera used in bELISA were selected from the aforementioned serum panel. Therein, five positive sera against ASFV corresponded to lane 1 to lane 5, and five ASFV-negative sera corresponded to first 5 numbered sera in fraction N1. Then, 40 positive ASFV sera and 98 negative ASFV sera were verified truly by Western blot.

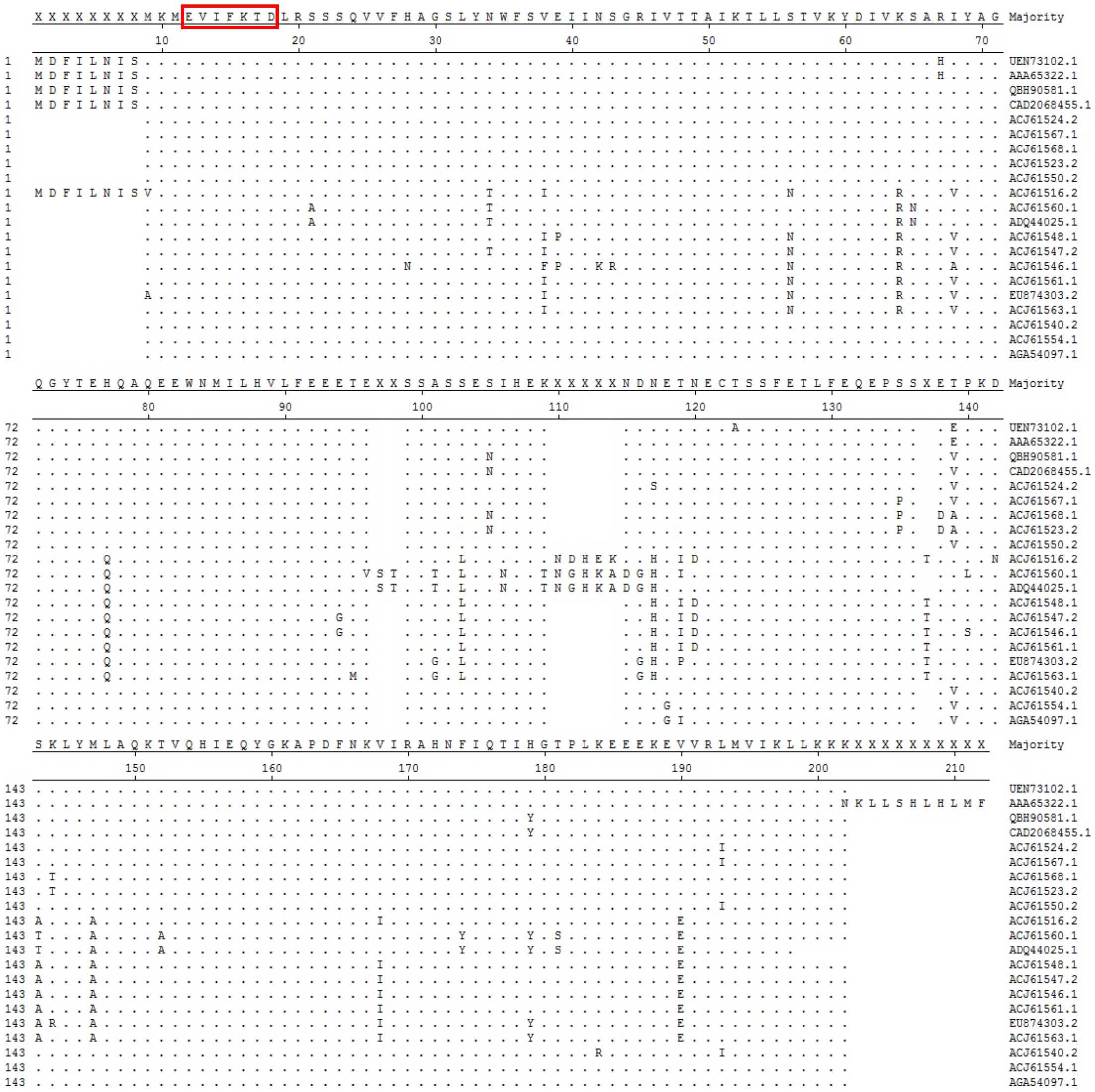

Figure S2. Sequence alignment of p30 proteins of 21 ASFV. Sequence alignment was performed with MegAlign. The consensus residues are denoted with a dot, while gap domains are denoted with blank. The amino acids on the top represent majority residues in identity position of p30. Epitope of amino acid 12–8 (EVIFKTD) recognized by mAb-2B4 is denoted with red box. The GenBank accession numbers of 21 ASFV p30 protein sequences contain the following: Genotype I (Pig/HeN/ZZ-P1/2021; UEN73102.1), Genotype I (BA71V; AAA65322.1), Genotype II (Pig/HLJ/2018; QBH90581.1), Genotype II (Georgia2007/1; CAD2068455.1), Genotype III (SPEC/257; ACJ61524.2), Genotype IV (RSA/04/3; ACJ61567.1), Genotype V (MOZ/1960; ACJ61568.1), Genotype VI (SPEC/265; ACJ61523.2), Genotype VII (SPEC/154; ACJ61550.2), Genotype VIII (PHW/88/1; ACJ61516.2), Genotype IX (KEN/05/1; ACJ61560.1), Genotype X (Ken09Tk.13/1; ADQ44025.1), Genotype XI (KAB/62; ACJ61548.1), Genotype XII (MZI/92/1; ACJ61547.2), Genotype XIII (SUM/1411; ACJ61546.1), Genotype XIV (NYA1/2; ACJ61561.1), Genotype XV (TAN/01/1; EU874303.2), Genotype XVI (TAN/03/1; ACJ61563.1), Genotype XIX (RSA/96/2; ACJ61540.2), Genotype XX (RSA/95/4; ACJ61554.1), and Genotype XXI (RSA/96/1/P; AGA54097.1).
